# Development of a Comprehensive Program for the Early Diagnosis and Treatment of Severe Infections in a Tertiary Hospital in Spain

**DOI:** 10.1093/ofid/ofaf532

**Published:** 2025-09-01

**Authors:** Guillermo Martín-Gutiérrez, José Molina, Carlos Martín-Pérez, Manuela Aguilar-Guisado, María Solla, Belén Ramos-Morán, Teresa Aldabó, Rosario Amaya-Villar, Adelina Gimeno, Pilar Egea, Rocío Álvarez-Marín, José Antonio Lepe, José Miguel Cisneros

**Affiliations:** Clinical Unit of Infectious Diseases, Microbiology and Parasitology, University Hospital Virgen del Rocío, Seville, Spain; Institute of Biomedicine of Seville (IBiS), University Hospital Virgen del Rocío/CSIC/University of Seville, Seville, Spain; Centro de Investigación Biomédica en Red de Enfermedades Infecciosas (CIBERINFEC), Madrid, Spain; Department of Health Sciences, Loyola Andalucía University, Sevilla, Spain; Clinical Unit of Infectious Diseases, Microbiology and Parasitology, University Hospital Virgen del Rocío, Seville, Spain; Institute of Biomedicine of Seville (IBiS), University Hospital Virgen del Rocío/CSIC/University of Seville, Seville, Spain; Centro de Investigación Biomédica en Red de Enfermedades Infecciosas (CIBERINFEC), Madrid, Spain; Clinical Unit of Infectious Diseases, Microbiology and Parasitology, University Hospital Virgen del Rocío, Seville, Spain; Institute of Biomedicine of Seville (IBiS), University Hospital Virgen del Rocío/CSIC/University of Seville, Seville, Spain; Centro de Investigación Biomédica en Red de Enfermedades Infecciosas (CIBERINFEC), Madrid, Spain; Clinical Unit of Infectious Diseases, Microbiology and Parasitology, University Hospital Virgen del Rocío, Seville, Spain; Clinical Unit of Infectious Diseases, Microbiology and Parasitology, University Hospital Virgen del Rocío, Seville, Spain; Institute of Biomedicine of Seville (IBiS), University Hospital Virgen del Rocío/CSIC/University of Seville, Seville, Spain; Critical Care Unit, University Hospital Virgen del Rocío, Seville, Spain; Critical Care Unit, University Hospital Virgen del Rocío, Seville, Spain; Clinical Unit of Infectious Diseases, Microbiology and Parasitology, University Hospital Virgen del Rocío, Seville, Spain; Institute of Biomedicine of Seville (IBiS), University Hospital Virgen del Rocío/CSIC/University of Seville, Seville, Spain; Clinical Unit of Infectious Diseases, Microbiology and Parasitology, University Hospital Virgen del Rocío, Seville, Spain; Clinical Unit of Infectious Diseases, Microbiology and Parasitology, University Hospital Virgen del Rocío, Seville, Spain; Institute of Biomedicine of Seville (IBiS), University Hospital Virgen del Rocío/CSIC/University of Seville, Seville, Spain; Centro de Investigación Biomédica en Red de Enfermedades Infecciosas (CIBERINFEC), Madrid, Spain; Clinical Unit of Infectious Diseases, Microbiology and Parasitology, University Hospital Virgen del Rocío, Seville, Spain; Institute of Biomedicine of Seville (IBiS), University Hospital Virgen del Rocío/CSIC/University of Seville, Seville, Spain; Centro de Investigación Biomédica en Red de Enfermedades Infecciosas (CIBERINFEC), Madrid, Spain; Department of Microbiology, University of Seville, Seville, Spain; Clinical Unit of Infectious Diseases, Microbiology and Parasitology, University Hospital Virgen del Rocío, Seville, Spain; Institute of Biomedicine of Seville (IBiS), University Hospital Virgen del Rocío/CSIC/University of Seville, Seville, Spain; Centro de Investigación Biomédica en Red de Enfermedades Infecciosas (CIBERINFEC), Madrid, Spain; Faculty of Medicine, University of Seville, Seville, Spain

**Keywords:** antimicrobial stewardship, DOOR-MAT analysis, rapid diagnostics, severe infections

## Abstract

**Background:**

To assess the impact of the rapid diagnosis and treatment of severe infections (rDTSI) program on diagnostic and clinical outcomes in patients with severe infections.

**Method:**

We conducted a pre–post quasi-experimental study evaluating patients with severe pneumonia or sepsis before (October 2019–February 2020) and after (March 2022–March 2023) rDTSI implementation. The program integrated rapid molecular diagnostics, a 24/7 laboratory workflow, and multidisciplinary training. Primary outcomes included time from clinical diagnosis to pathogen-directed therapy and targeted therapy within 48 h. Secondary outcomes assessed antimicrobial appropriateness (DOOR-MAT), length of stay, and mortality.

**Results:**

The rDTSI program significantly reduced the median time to pathogen-directed therapy in pneumonia (48.8 vs 23.6 h, *P* < .001) and increased targeted therapy within 48 h (36.17% to 58.14%, *P* = .049). Hospital stays decreased (38.9 to 22.2 days, *P* < .001). In sepsis, diagnostic times (19.4 vs 18.1 h, *P* = .028) and DOOR-MAT scores (80.4 vs 88.0, *P* = .024) improved, while other clinical outcomes remained unchanged.

**Conclusions:**

The rDTSI program accelerated microbiological diagnosis, optimized antimicrobial therapy, and improved hospital efficiency in severe infections. These findings support integrating rapid diagnostics into antimicrobial stewardship programs to enhance severe infection management.

## INTRODUCTION

Severe infections are characterized by rapid progression, poor prognosis and diagnostic difficulty, the most common of which include bloodstream infections potentially associated with sepsis or septic shock [1], as well as severe community-acquired pneumonia (CAP) and ventilator-associated pneumonia (VAP) [2, 3]. In such cases, initiating empirical antimicrobial therapy is recommended immediately after obtaining microbiological diagnostic samples. However, inadequate empirical therapy has been associated with worse clinical outcomes [4, 5], highlighting the critical importance of early pathogen identification to guide appropriate therapy.

Traditionally, organism identification and antimicrobial susceptibility testing have turnaround times of 48 hours or more. Moreover, in patients with resistant pathogens there is an increased risk that empirical antimicrobial treatment may be ineffective, leading to higher mortality rates [6, 7]. Given the above, rapid microbiology diagnosis could have a critical impact in preventing delays in starting appropriate treatments. New molecular techniques have been applied in routine workflow to provide rapid pathogen identification and resistance genes or resistance profiles in patients with sepsis and pneumonia [8, 9]. Recent studies have demonstrated that the implementation of rapid molecular methods increased the appropriateness of empirical treatment [10], and decreased time to first antibiotic change [11]. However, several studies did not find differences in cure [12], length of stay (LOS) [13], or mortality [14, 15].

The purpose of this study is to assess the impact of a comprehensive program for the rapid microbiological diagnosis and management of severe infections—known as the Rapid Diagnosis and Treatment of Severe Infections (rDTSI) program—implemented within the framework of a well-established antimicrobial stewardship program (ASP) in a tertiary hospital setting. In addition to evaluating clinical and microbiological outcomes associated with the intervention, we also aimed to explore patient-related factors that may influence prognosis and potentially modify the response to early diagnostic and stewardship strategies.

## MATERIAL AND METHODS

### Study Design

This single-center, pre–post quasi-experimental study was performed at the University Hospital Virgen del Rocio (Seville, Spain), a teaching hospital providing a tertiary-care service with 1113 beds (including 62 adult intensive care unit beds), with active solid-organ and hematopoietic stem-cell transplantations programs. In this study, we compared the outcomes of patients with severe infections (sepsis and pneumonia) before (October 2019–February 2020) and after (March 2022–March 2023) the implementation of the rDTSI program in our hospital. There is a time gap between the preintervention and postintervention periods, as we intentionally excluded the SARS-CoV-2 pandemic phase (March 2020–February 2022) to avoid the profound disruptions in hospital operations, antimicrobial prescribing, and diagnostic priorities that could confound the results.

Clinical, demographic, and microbiological data were extracted from the electronic health record (EHR) and microbiology information systems using a standardized data collection form. When necessary, additional information was retrieved through manual chart review to complete missing or unclear entries. Patient consent was not required as this study was conducted using anonymized data obtained during routine clinical care, in accordance with the standards currently applied in Spain. The study protocol was reviewed and approved by the management of the University Hospital Virgen del Rocío (Seville, Spain). No additional procedures or interventions outside standard care were performed.

### Participants

We included consecutive adult patients (≥18 years) with clinical suspicion of sepsis [1], severe CAP, VAP, or hospital-acquired pneumonia who underwent microbiological sampling. In the preintervention period, as these electronic profiles were not yet available, patient identification was based on the clinical presentation consistent with severe infection and the receipt of corresponding microbiological samples in the laboratory. During the intervention period, patient inclusion was based on the activation of 1 of 2 predefined diagnostic profiles—“sepsis profile” or “pneumonia profile”—implemented within the electronic medical record as part of the rDTSI program. These profiles were accessible 24/7 across all clinical units and, once activated by the treating physician, triggered immediate notification to the microbiology laboratory and infectious diseases team to ensure prioritized processing and follow-up.

Inclusion criteria required activation of 1 of the 2 profiles and collection of at least 1 respiratory or blood sample for microbiological analysis. The final diagnosis of pneumonia was established retrospectively using prespecified clinical, radiological, and microbiological criteria derived from established guidelines and expert reviews, including the ATS/IDSA guideline [16] and other published sources (see Supplementary Table 1).

### Procedures

The rDTSI program was implemented through a standardized digital activation process embedded in the electronic medical record, which guided clinicians through syndrome-specific diagnostic pathways. Upon activation of the sepsis or pneumonia profile, the system generated predefined recommendations for sample collection (eg, blood cultures and respiratory specimens) and triggered microbiological workflows including Gram staining, culture, syndromic multiplex PCR, and antigen detection as appropriate. Test results were continuously monitored by the microbiology laboratory and communicated in real time to the Infectious Diseases team, who provided targeted stewardship interventions. This integrated approach was available 24/7 and adopted across all relevant hospital departments. In addition, the program was carried out within the framework of an ASP called the Institutional Program for the Optimization of Antimicrobial Therapy (PRIOAM) that started in the hospital in 2011 and has been successful in improving antimicrobial use and reducing the incidence of nosocomial infections due to multidrug-resistant microorganisms [17].

The activity of the rDTSI program consisted of 3 components (Supplementary Figure 1): (1) educational and training programs for physicians, nurses, and laboratory technicians from the involved clinical units, delivered through in-person clinical sessions and interactive workshops conducted by a group of clinical experts designated by the PRIOAM team; (2) specific training for laboratory technicians and clinical microbiologists, emphasizing the importance of urgent sample processing, correct sample handling, and the performance and interpretation of molecular techniques; and (3) training sessions aimed at all participating clinical services, focused on the interpretation of rapid molecular test results and the optimization of antimicrobial treatment according to PRIOAM guidelines. These sessions were complemented by regular feedback reports and individualized case-based advisory meetings with each unit or service, based on their submitted diagnostic requests and the microbiological results obtained. In addition, a dedicated transport system was established using red bags labeled with the program's logo to ensure rapid identification and prioritization of samples in the microbiology laboratory (Supplementary Figure 2). All educational content and guidance materials were made available on the PRIOAM website to facilitate access by clinical staff (https://www.guiaprioam.com/section/programa-para-la-prevencion/).

### Microbiology Workflow

Microbiological workflows differed between the preintervention and intervention periods in terms of laboratory operating hours, respiratory sample processing, and turnaround time. During the preintervention phase, blood cultures were processed daily from 08:00 to 22:30, with preliminary results communicated within 1 h of positivity detection. In the intervention period, laboratory operations were expanded to a 24/7 schedule, ensuring continuous diagnostic activity.

For patients with suspected pneumonia, both bronchial aspirates and bronchoalveolar lavages (BAL) were accepted in the preintervention period. In contrast, only BAL samples were processed in the intervention period for molecular diagnostic testing. The availability of molecular testing for respiratory pathogens also expanded from limited hours (08:00–22:30) to 24/7 during the intervention phase.

Patients were assigned to either the pneumonia or sepsis cohort according to the diagnostic profile activated in the electronic medical record. In cases where both conditions overlapped, classification was based on the profile selected by the clinician, complemented by the predominant clinical syndrome and the type of specimen submitted.

**Figure 1.**
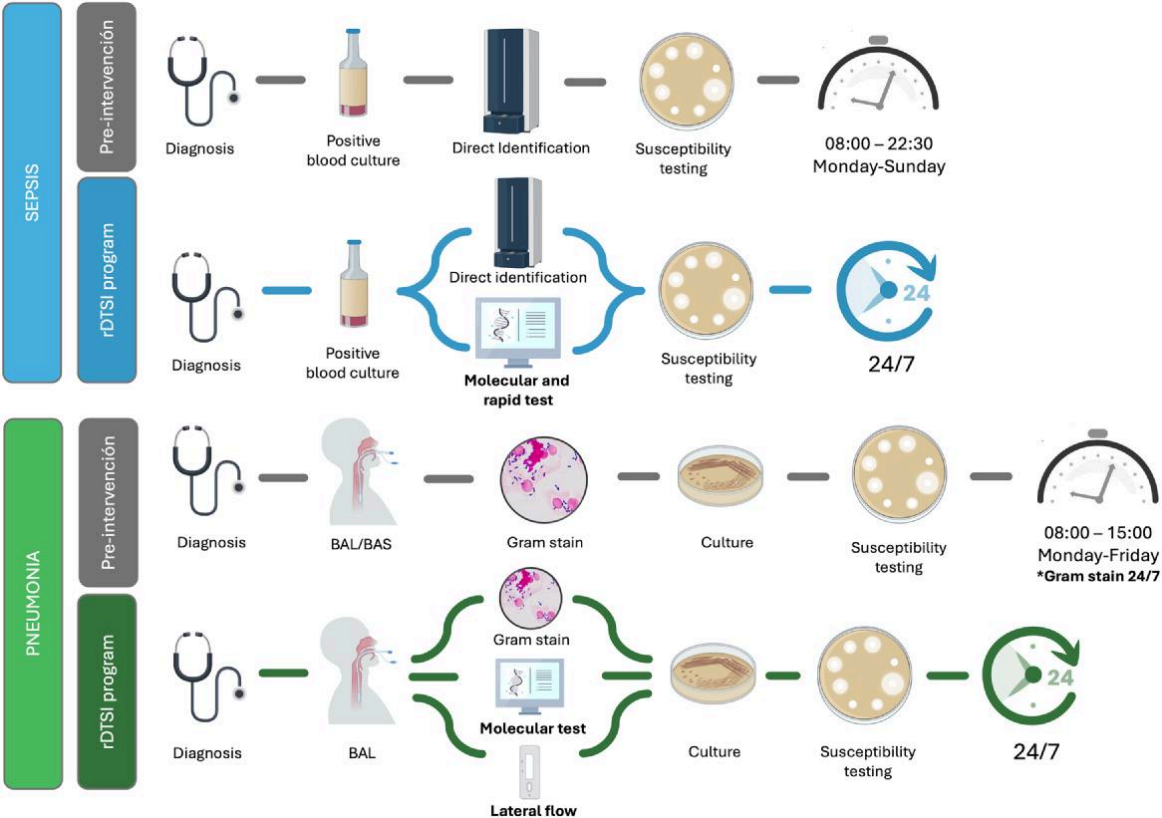
Workflows for the microbiological diagnosis of severe pneumonia and sepsis in the preintervention and intervention periods. This figure has been designed using images from Flaticon (www.flaticon.com), Freepik (https://www.freepik.es/), and BioRender (https://BioRender.com/60nyi0z).

The microbiological diagnostic workflows implemented during the preintervention and intervention periods are summarized in Figure 1. In both study periods, direct identification from positive blood cultures using MALDI-TOF MS was routinely performed as part of the standard microbiological workflow. Although not universally adopted in clinical laboratories, this technique likely contributed to shorter turnaround times in both cohorts. Additionally, multiplex molecular testing was performed using commercially available syndromic panels targeting a broad range of bacterial, viral, and fungal pathogens in blood and lower respiratory tract specimens. Full methodological details, including the specific molecular tests and targets (Supplementary Table 1), as well as complete microbiological results, are provided in Supplementary Material 2.

### Outcomes

The 2 primary outcomes were: (1) pathogen-directed treatment based on a microbiological test result within 48 h. Pathogen-directed treatment was defined as the administration of at least 1 antimicrobial agent with in vitro activity against the identified pathogen. This included both escalation (initiation of appropriate coverage when the empiric regimen was inactive) and de-escalation (narrowing of the initial regimen when it included unnecessarily broad coverage). Time to pathogen-directed treatment was calculated from clinical diagnosis to the first administration of a targeted agent; and (2) time from clinical diagnosis to pathogen-directed treatment. The time of clinical diagnosis was defined as the first documented mention of the infectious syndrome (sepsis or pneumonia) in the electronic medical record by the treating physician, including progress notes, diagnostic assessments, or treatment decisions. Two investigators (B.R.-M. and M.S.) assessed whether and when a patient received pathogen-directed antimicrobial treatment based on a microbiological test result. In case of disagreement, a third investigator (G.M.-G.) arbitrated. For the purpose of this study, the final microbiological report was defined as the time point at which antimicrobial susceptibility testing results from conventional culture-based methods became available in the electronic medical record. Additionally, LOS in days and mortality (7, 14, and 28 days) and DOOR-MAT score.

### DOOR-MAT Score

**Figure 2.**
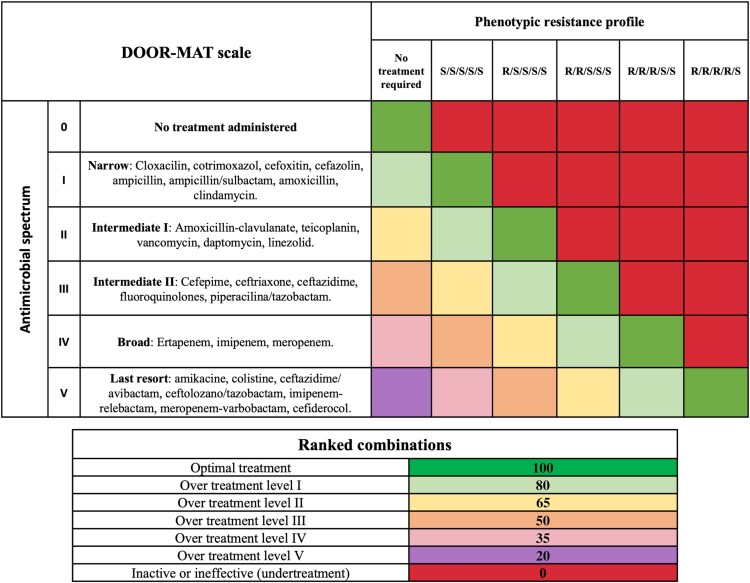
DOOR-MAT matrices developed based on local institutional infectious diseases epidemiology and prescribing practices (PRIOAM), and the credit scoring system assigned to each of these categories.

To evaluate potential differences in antimicrobial prescribing based on the intervention, DOOR-MAT matrices were developed [18] (Figure 2). The analysis included only patients with positive microbiological results. The DOOR-MAT score was evaluated in 2 ways: (1) by analyzing the treatment chosen after the issuance of microbiological reports, and (2) at different time points (12, 24, and >48 h) from the clinical diagnosis of the infectious syndrome. In cases of combination therapies (2 or more antibiotics), the DOOR-MAT score was calculated integrating both targeted (required) and not-required treatments. The formula used was:


$$\mathrm{DOOR}\text{-}\mathrm{MAT}\;\mathrm{Score}=\frac{\mathrm{DOOR}\text{-}\mathrm{MAT}\;\mathrm{Scor}e_{\mathrm{targeted}}+\sum_{i=1}^{n}\;\mathrm{DOOR}\text{-}\mathrm{MAT}\;\mathrm{Scor}e_{\mathrm{not}\text{-}\mathrm{required},i}}{n+1}$$

DOOR-MAT score _targeted_ represents the score assigned to the antimicrobial with the highest DOOR-MAT score for the causative pathogen; $\overset{n}{\underset{i=1}{\sum }}\;$ DOOR-MAT score _not-required_ is the sum of all not-required scores, with n indicating the number of not-required antimicrobials, which can range from 1 to X. If 2 or more treatments had the same score, the most appropriate option was selected as targeted therapy based on PRIOAM guidelines, while the remaining treatments were considered as not-required treatments. In cases of mixed bacterial infections, the DOOR-MAT score was assigned based on the pathogen requiring the broadest spectrum or considered most resistant. For coinfections involving bacterial and nonbacterial pathogens (eg, fungi or viruses), only the bacterial component was considered, as the DOOR-MAT framework evaluates antibacterial therapy.

Interpretation of the DOOR-MAT score: Higher scores indicate more desirable prescribing decisions, balancing appropriate coverage with antimicrobial stewardship. For example, a patient with *Escherichia coli* treated with ceftriaxone would receive a higher score than one treated with meropenem, assuming both are active against the pathogen. Conversely, a patient with *P. aeruginosa* treated with an inactive agent or excessively broad combination would receive a lower score. This framework thus allows assessment of both the adequacy and parsimony of antimicrobial use in clinical decision-making.

### Statistical Analysis

Categorical variables were compared using χ^2^ or Fisher's exact test. Continuous variables were analyzed using Student's *t*-test or Mann–Whitney *U* test, depending on normality. Time-to-event data (eg, mortality) were analyzed via Cox proportional hazards models. Nonlinear associations and variable interactions were explored using random survival forests (RSF). Variables with >10% missing data were excluded from multivariable models. A *P*-value <.05 was considered statistically significant. Statistical analyses were performed using R (v4.2.1).

To account for baseline differences between study periods and to identify independent predictors of adverse outcomes, we performed multivariable analyses using both Cox models and RSF. These models were applied separately to the pneumonia and sepsis cohorts, with mortality and LOS as primary outcomes. Cox models were built using a stepwise approach, including clinically relevant variables and those with *P* < .1 in univariable analysis. RSF was used to rank variable importance using permutation-based metrics and to explore nonlinear relationships and interactions. Model performance was evaluated using Brier scores and time-dependent AUC.

To achieve the most accurate prediction of mortality possible, we leveraged the complementary strengths of both approaches. RSF offers superior predictive performance and can handle complex data structures, but typically requires a larger number of events for optimal training. In contrast, Cox regression is well suited for datasets with fewer events and provides more interpretable hazard ratios. By combining these methods, we aimed to balance predictive accuracy with clinical interpretability.

These analyses were intended not only to adjust for potential confounders when comparing study periods, but also to identify which patient characteristics were independently associated with adverse outcomes, potentially modifying the effect of the intervention

## RESULTS

### Patient Characteristics

In this study, 120 patients with severe pneumonia were included, 59 (49.2%) in the preintervention period and 61 (50.8%) in the intervention period. Regarding patients with sepsis, 218 were included, of which 110 (50.5%) were included in the preintervention period and 108 (49.5%) in the intervention period. Total patients included 117 females (34.6%) and were 221 males (65.4%) men, with a median age of 66 (56–74). Patient characteristics are summarized in Table 1. Detailed microbiological findings are provided in Table 2 and Supplementary Material 2. The 28-day mortality was 25% (30 of 120) for patients with pneumonia and 17.9% (39 of 218) for those with sepsis.

**Table 1.** Baseline Characteristics for all Included Patients

	No. (%)					
Characteristic	Pneumonia			Sepsis		
	Preinterv	Intervention	*P-*value	Preinterv	Intervention	*P-*value
	(*n* = 59)	(*n* = 61)		(*n* = 110)	(*n* = 108)	
Age, mean (SD)	57.59 (15.1)	60.87 (11.8)	1	69.33 (12.7)	65.75 (15.7)	.098
Sex						
Female	21 (35.6)	14 (22.9)	.128	40 (36.4)	42 (38.89)	.7
Male	38 (64.4)	47 (77)		70 (63.6)	66 (61.1)	
Current smoker	26 (44)	40 (65.6)	**.017**	22 (20.2)	48 (44.4)	**<.001**
Alcohol consumption	13 (22)	13 (21.3)	.923	12 (11)	21 (19.4)	.083
Antibiotic within past m	14 (23.7)	27 (44.3)	**<.001**	13 (11.9)	26 (24.1)	**.02**
Charlson index (median)	3	4	**.002**	5.3	4.6	**.009**
Comorbidities						
Hypertension	29 (49.1)	32 (52.5)	.717	72 (66.1)	52 (48.1)	.**007**
Respiratory disease	9 (15.2)	12 (19.7)	.524	6 (5.5)	20 (18.5)	.**003**
CVD	7 (11.9)	3 (4.9)	.201	28 (28)	21 (19.4)	**<.001**
Kidney disease	5 (8.5)	9 (14.7)	.284	15 (13.8)	18 (16.7)	.551
Liver disease	1(1.7)	4 (6.6)	.364	16 (14.7)	7 (6.5)	.049
Diabetes	15 (25.4)	16 (26.2)	.92	28 (25.9)	31 (28.7)	.647
Cancer	5 (8.5)	28 (46)	**<.001**	44 (40.4)	46 (42.6)	.739
Neutropenia	2 (3.4)	7 (11.5)	**<.001**	9 (8.2)	15 (13.9)	.**001**
COVID	0 (0)	16 (26.2)	**<.001**	0	0	…
Chronic treatments						
ACE inhibitors	18 (30.5)	21 (34.4)	…	40 (36.4)	39 (36.1)	
Glucocorticoid	4 (6.8)	23 (37.7)	**<.001**	5 (4.6)	19 (17.6)	.**002**
Warfarin	14 (23.7)	11 (18)	…	7 (6.4)	19 (17.6)	
Acquisition						
Community	9 (15.2)	28 (45.9)	**<.001**	72 (65.4)	75 (69.4)	.529
Nosocomial	50 (84.7)	33 (54.1)		38 (34.5)	33 (30.6)	
Type of pneumonia						
sCAP (CURB65 ≥ 3)	10 (17%)	24 (39.3%)	.**006**	…	…	…
Nosocomial pneumonia	49 (83%)	37 (60.6%)		…	…	…
VAP	37 (62.7%)	15 (24.6%)	**<.001**	…	…	…
Sepsis syndrome						
Sepsis	…	…	…	93 (84.5)	80 (74.1)	.056
Septic shock	…	…	…	17 (15.4)	28 (25.9)	

Bold values indicate statistically significant differences (in the *P*-value).

Preinterv, preintervention period; CVD, cardiovascular disease; sCAP, severe community-acquired pneumonia; VAP, ventilator-associated pneumonia; IP, immunocompromised patient.

**Table 2.** Microbiological Findings From the Study

Pneumonia				Bacteremia			
Type of infection		Preinterv	Intervention	Type of infection		Preinterv	Intervention
		*n* (%)	*n* (%)			*n* (%)	*n* (%)
Bacterial	…	…	…	Bacterial		…	…
* P. aeruginosa*		7 (17.5)	6 (15)	…	*E. coli*	17 (26.56)	16 (32.65)
* S. aureus*		6 (15)	5 (12.5)	…	*S. aureus*	10 (15.62)	3 (6.12)
* E. coli*		4 (10)	3 (7.5)	…	*K. pneumoniae*	9 (10.93)	10 (20.4)
Other		12 (30)	15 (37.5)	…	*P. aeruginosa*	5 (7.81)	5 (10.2)
Fungal	*…*	…	…	…	*E. cloacae*	3 (4.68)	1 (2.04)
*Aspergillus spp.*		3 (7.5)	5 (12.5)	…	Other	13 (20.31)	10 (20.4)
*A flavus*^a^		0 (0)	1 (2.5)	Fungal		…	…
*A. fugmigatus*		2 (5)	1 (2.5)	…	*Candida* spp.	2 (3.12)	3 (6.12)
*A. lentulus*		0 (0)	1 (2.5)	Mixed infections		…	…
*A. niger*		1 (2.5)	1 (2.5)	…	2 microorganisms	5 (7.81)	1 (2.04)
*A. terrus*		0 (0)	1 (2.5)	…	…	…	…
Viral		…	…	…	…	…	…
Influenza		6 (15)	3 (15)	…	…	…	…
Others		0 (0)	2 (5)	…	…	…	…
Mixed infections		…	…	…	…	…	…
2 microorganisms		8 (20)	3 (7.5)	…	…	…	…
3 microorganisms		0 (0)	3 (7.5)	…	…	…	…

Preinterv, preintervention period.

^a^Coinfection with *Rhizopus orizae*.

### Primary Outcomes

In the pneumonia cohort, pathogen-directed treatment within 48 h increased from 36.2% (17/47) in the preintervention period to 58.1% (25/43) in the intervention period (*P* = .049). The median time from clinical diagnosis to initiation of pathogen-directed therapy was significantly reduced from 48.8 h (IQR 30.9–83.6) to 23.6 h (IQR 8.9–37.5) (Table 3). In patients with sepsis, no significant differences were observed in the proportion receiving pathogen-directed treatment within 48 h (46.9% preintervention vs 53.7% intervention, *P* = .43), nor in the median time to initiation of pathogen-directed treatment (40.2 h [IQR 28.1–66.8] vs 38.5 h [IQR 21.4–60], respectively) (Table 3).

**Table 3.** Patients Receiving Pathogen-directed Treatment After Microbiology Reports (Med [IQR])

Outcomes	Pneumonia			Sepsis		
	Preintervention	Intervention	*P-*value	Preintervention	Intervention	*P-*value
Event-time outcomes						
Time from clinical diagnosis to pathogen-directed treatment (h)	48.8 (30.9–83.6)	23.6 (8.9–37.5)	**<.001**	40.2 (28.1–66.8)	38.5 (21.4–60)	.106
Time from specimen collection to pathogen-directed treatment (h)	29.1 (14.2–39.9)	19.6 (4.5–24.9)	.**049**	30.6 (28.1–66.8)	26.7 (11.9–37.5)	.443
Length of hospital stay (d)	38.9 (19.9–69.6)	24.8 (13.4–40.3)	**<.001**	10.8 (6.9–21.8)	11.9 (7.9–19.4)	.815
Preinterv: preintervention period.	…	…		…	…	

Bold values indicate statistically significant differences (in the *P*-value).

When analyzing the response times in the microbiology laboratory before and after the implementation of the rDTSI program for patients with severe pneumonia, we observed a significant decrease in the time from specimen collection to RDT results report (median time 21.7 [CI 95% 1.0–51.3] vs 3.0 [CI 95% 2.0–4.4] h, *P* = .004), as well as in the time required to achieve microorganism identification (44.1 [CI 95% 23.6–58.4] vs 24.9 h [CI 95% 3.6–59.8], *P* = .008) (Table 4). Moreover, the time from clinical diagnosis to pathogen-directed treatment was significantly reduced in patients with pneumonia (48.8 [CI 95% 30.9–83.6] vs 23.6 [CI 95% 8.9–37.5] h, *P* < .001; Table 3). Similarly, in the sepsis cohort, the time from specimen collection to microorganism identification was significantly shortened in the intervention group (19.4 [CI 95% 16.3–30.7] vs 18.1 [CI 95% 13.2–23.6] h, *P* = .028) (Table 4), reflecting the positive impact of the program on diagnostic turnaround times.

**Table 4.** Comparison of Time to Microbiology Results (Hours) in Preintervention and Intervention Periods (Med [IQR])

	Pneumonia			Sepsis		
Microbiology results	Preinterv	Intervention	*P*-value	Preinterv	Intervention	*P-*value
Time from specimen collection to RDT result^a^	21.7 (1.0–51.3)	3.0 (2.0–4.4)	.**004**	…	…	…
Time from specimen collection to microorganism identification	44.1 (23.6–58.4)	24.9 (3.6–59.8)	.**008**	19.4 (16.3–30.7)	18.1 (13.2–23.6)	.**028**
Time from specimen collection to final bacterial reports^b^	75.7 [59.5–95.0]	59.2 [46.6–98.1]	.168	42.2 [38.3–59.3]	41.0 [33.9–58.3]	.598
Time from specimen collection to final microbiological reports^c^	73.6 (55.9–93.8)	89.6 (47.5–167.3)	.2125	42.9 (38.3–58.5)	41.3 (34–58)	.626

Bold values indicate statistically significant differences (in the *P*-value).

Preinterv, preintervention period.

^a^In this analysis, all the PCRs or molecular tests performed (rapid diagnosis test [RDT]) in patients with pneumonia are included.

^b^Final microbiology report: this report included the bacterial identification as well as the antimicrobial susceptibility testing.

^c^Final microbiology report: this report included the microorganism identification (bacteria and fungi) as well as the antimicrobial/antifungal susceptibility testing.

### DOOR-MAT Analysis

**Figure 3.**
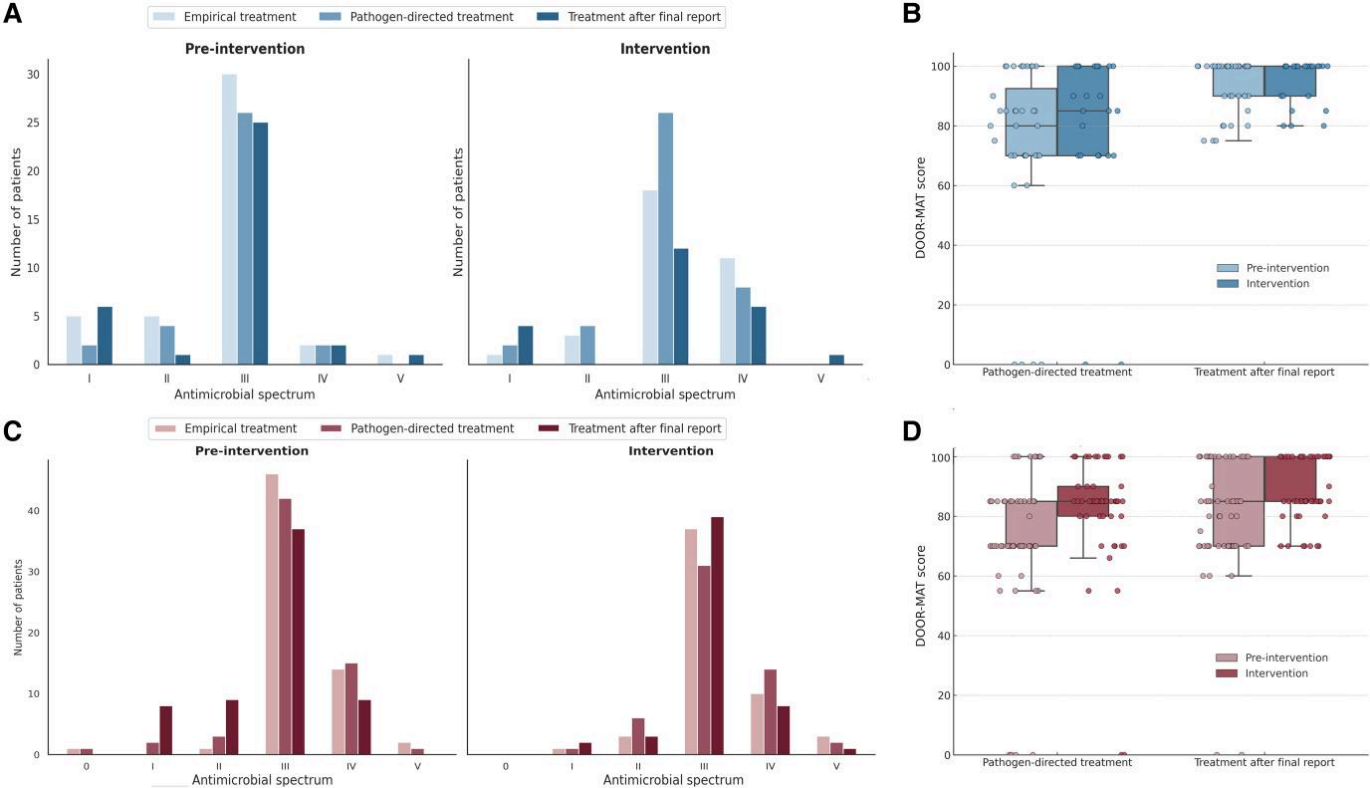
Impact of a rapid diagnostic intervention on antimicrobial spectrum and clinical decision quality (DOOR-MAT score) in patients with severe pneumonia and sepsis. (*A*) Distribution of empirical, pathogen-directed, and final treatments according to antimicrobial spectrum (categories 0–V) in patients with severe pneumonia, before and after the intervention. (*B*) Comparison of DOOR-MAT scores in the same pneumonia cohort for pathogen-directed treatment and treatment after final microbiology report, preintervention and postintervention. (*C*) Distribution of empirical, pathogen-directed, and final treatments by spectrum in patients with sepsis, before and after the intervention. (*D*) Comparison of DOOR-MAT scores in the sepsis cohort for pathogen-directed and final treatment, preintervention and postintervention. Boxplots show median, interquartile range (IQR), and full range. Individual patient data points are overlaid. The DOOR-MAT score reflects the clinical desirability of the chosen regimen, integrating efficacy and potential toxicity.

We observed no significant differences in the DOOR-MAT spectrum for the empirical treatment between the preintervention and intervention periods in pneumonia (Figure 3; Supplementary  Table 2). We observed an increase in the DOOR-MAT score in the intervention period compared with the preintervention period (74.2 [CI 95% 64.5–83.9] vs 81.2 [CI 95% 34.8–91.5], *P* = .213), though it did not achieve statistical significance. After the final microbiology report, no significant differences in the DOOR-MAT score were observed (93.7 [CI 95% 90.7–96.6] vs 94.1 [CI 95% 90.1–97.3], *P* = .558).

In sepsis, we observed no significant differences for empirical treatments between the 2 periods analyzed (Table 5), nor in the number of combined treatments (35% vs 37%, *P* = .701). After pathogen identification, we observed an increase in the DOOR-MAT score in the intervention period (71.2 [CI 95% 65.2–77.202] vs 81.2 [CI 95% 75.7–86.6], *P* = .001), without observing significant differences in the spectra used. After the final microbiology report, the DOOR-MAT score was significantly higher in the intervention period (80.4 [CI 95% 75.6–85.2] vs 88 [CI 95% 85.1–90.9], *P* = .024). This improvement suggests more appropriate antimicrobial selection, better aligned with pathogen susceptibility, and overall higher prescribing quality as captured by the DOOR-MAT score.

**Table 5.** Mortality (7, 14, and 28 Days) *N* (%)

Syndrome	Mortality *N* (%)								
	7 D			14 D			28 D		
	Preinterv	Intervention	*P*-value	Preinterv	Intervention	*P*-value	Preinterv	Intervention	*P*-value
Severe pneumonia	3 (5.08%)	8 (13.11%)	0.516	8 (15.68%)	14 (22.95%)	.869	9 (15.25%)	21 (34.42%)	.164
Sepsis	17 (15.45%)	10 (9.25%)	0.333	19 (17.27)	14 (12.96)	.367	23 (20.9%)	16 (14.81%)	.319

Preinterv, preintervention period.

### Length of Stay and Clinical Outcomes

In patients with severe pneumonia, we observed a significant decrease in length from a median of 38.9 days to 22.2 days (*P* < .001). In the multivariable analysis for potential factors associated with LOS, the final model indicated a positive association for patients with VAP (375.8, *P* = .018) and drinking history (465.8, *P* = .009), indicating that these factors were linked to prolonged hospitalization. Conversely, the use of a rapid molecular diagnostic technique was associated with shorter LOS (−407.9, *P* = .021), suggesting that its implementation may contribute to a reduction in hospitalization duration (Supplementary Material 1 and Supplementary Table 3). Regarding mortality, no significant differences were observed when they were compared (Table 5), although we observed an overall increase in the intervention cohort (20.3% vs 39.3%, *P* = .023). In the sepsis cohort, we found no significant differences in length of hospital stay between the preintervention and intervention periods (9.1 vs 10.62 days, *P* = .231), nor for mortality (Table 5).

### Cox and RSF Analysis

We conducted exploratory Cox regression and RSF analyses to identify independent predictors of 28-day mortality in each cohort. In patients with pneumonia, fungal infections, influenza-related admissions, and a higher Charlson index were significantly associated with increased risk of death. In the sepsis cohort, mortality was more likely in patients with negative blood cultures, absence of antimicrobial adjustment after microbiological results, lower DOOR-MAT scores, higher Charlson index, and longer delays in initiating empirical therapy. The RSF model supported these findings and ranked similar variables as most influential. Full model outputs are provided in Supplementary  Tables 3 and 4 and Figures 3–5.

## DISCUSSION

The results of this study suggest that the implementation of the rDTSI program was associated with a reduction in the time to microbiological results, the time to initiation of targeted antimicrobial therapy, and the length of hospital stay in patients with severe pneumonia. In patients with sepsis, the program was associated with a shorter time to microbiological diagnosis and improvements in the quality of antimicrobial prescribing, as measured by the DOOR-MAT score. Specifically, the increase in DOOR-MAT scores during the intervention period (from 80.4 to 88.0, *P* = .024) suggests more appropriate antibiotic choices, with better alignment to pathogen susceptibility profiles. To our knowledge, this is the first study to evaluate the impact of a comprehensive program for rapid diagnosis and management of severe infections—including syndromic panels, training measures, and logistical improvements—within the framework of an established ASP. While these findings support the potential benefit of integrating rapid diagnostics into clinical workflows, we acknowledge that the quasi-experimental design precludes definitive causal conclusions, and prospective randomized studies would be needed to confirm these associations.

The implementation of the rDTSI program led to a significant reduction in the time required for identification of the causative agent as well as targeted therapy in patients with severe pneumonia. The accomplishment of this approach is concordant with previous studies where the implementation of RDT for the diagnosis of patients with severe pneumonia showed a significant improvement of the quality of antibiotic prescriptions [19–23]. In addition, in our study, we observed a significant decrease in the length of hospital stay in the intervention cohort. Only the study performed by Kluczna et al [20] showed a decrease in LOS of 5.6 days in hospital in patients with VAP, while no such difference was observed in other previous studies. Our results may suggest that a rapid diagnosis of the etiological agent, as well as a reduction in time to pathogen-directed treatment, may be associated with early recovery, reducing the LOS. However, there is a higher proportion of patients with VAP in the preintervention period, which are often associated with longer periods of hospitalization compared with other types of severe pneumonia. Therefore, these findings should be interpreted with caution.

Although the implementation of this program has led to significant improvements in the diagnosis and treatment of patients with severe pneumonia, a nonsignificant increase in mortality was observed. This may be due 2 main factors: (1) in the intervention phase, we included more patients with filamentous fungal infections, which are usually associated with higher mortality [24], and (2) a higher proportion of immunocompromised and patients with cancer were included in the intervention phase. Immunocompromised patients experience more severe complications that progress to severe pneumonia and worse outcomes [25, 26]. Consistent with existing evidence, our results confirmed that fungal infections, elevated Charlson index and cancer were significantly associated with an increased risk of mortality, which could explain the higher mortality in the intervention period.

In patients with bacteremia, we observed a significant reduction in the time to etiological diagnosis, as well as an improvement in the quality of antimicrobial treatment for pathogen-directed treatment. These findings suggest that the implementation of a rapid diagnosis and treatment program for sepsis may act as a reinforcement for the positive outcomes previously achieved through our ASP [17, 27]. In addition, our findings revealed that patients without an etiological diagnosis (negative blood culture) were associated with higher mortality. Recent evidence has shown that patients with negative blood cultures are more likely to receive inappropriate treatment, and that persistent inappropriate treatment beyond the second day is linked to increased mortality [28].

In contrast to previous findings, we found no significant differences in survival in patients with sepsis after implementation of the rDTSI program. Previously, 2 meta-analyses [29–31] have shown that implementation of RDT in bacteremia diagnosis significantly improved pathogen-directed treatment, as well as patient outcome, always in association with ASP. These studies compared rapid tests (MALDI-TOF or PCRs) against the traditional method (Gram stain and culture). In our case, in the preintervention period we already routinely used the MALDI-TOF as a RDT daily from 08:00 to 22:30. The implementation of the syndromic panel and increasing the working time to a 24/7 laboratory did not allow us to observe any improvement in patient outcome. The low resistance rates observed during the study period may limit the utility of molecular studies for the rapid detection of genetic markers associated with antimicrobial resistance. Moreover, while only 6 blood cultures had a positive result from 22:30 to 08:00 (data not shown), and a reduction to obtain the etiological diagnosis from 19.4 to 18.1 h were observed, further studies would therefore be needed to demonstrate the real impact of reporting positive blood cultures 24/7. Nonetheless, our findings may still support the implementation of similar rapid diagnostic and stewardship programs in healthcare settings without MALDI-TOF access or with limited laboratory hours. In such contexts, where diagnostic turnaround times are longer, the clinical benefits of syndromic molecular panels and coordinated workflows may be more pronounced, potentially leading to improved patient outcomes.

This study has several limitations. First, the pre–post quasi-experimental study design inherently lacks randomization, which may result in imbalanced populations between the 2 study periods. In our case, this limitation is particularly evident in the pneumonia cohort, where differences in baseline characteristics could reduce the validity of direct comparisons of prognostic variables. To address this issue, we performed a Cox and RSF analysis to account for these imbalances and provide a more robust assessment of outcomes [32–34]. Second, the single-center design limits generalizability. Third, this study has not included a cost-effectiveness analysis. Fourth, although inclusion criteria were broad and included all main types of severe pneumonia, we cannot conclude which types of pneumonia would benefit the most from this intervention. The small number of patients makes statistical analysis of the subgroups not possible. Fifth, adjudicators evaluating whether patients received pathogen-directed treatment were not blinded to the study period, as this information was inherently linked to the data extracted from electronic medical records. Although predefined criteria were applied and assessments were conducted independently by 2 investigators, with arbitration by a third in case of discrepancies, the absence of blinding could have introduced classification bias. Sixth, the real-time involvement of Infectious Diseases specialists, although central to the success of the intervention, requires significant resource investment and institutional support, which may limit immediate implementation in settings without dedicated 24/7 ID coverage. Finally, the DOOR-MAT scale was defined by the research team and is not a standardized tool, so the results could differ if alternative scales were applied.

In conclusion, our study illustrates that the multidisciplinary care program for the rapid diagnosis and comprehensive management of severe infections was associated with a reduction in time to change in antibiotics, microbiological diagnosis, as well as reduced LOS in patients with severe pneumonia. For patients with sepsis, we observed a significant improvement in the time necessary to etiological diagnosis, as well as on the quality of the treatment analyzed with DOOR-MAT. Prospective randomized controlled trials are needed to confirm these results, identifying patients that would most benefit from this approach. These findings highlight not only the potential of coordinated diagnostic and stewardship interventions, but also the need to identify patient subgroups who may benefit most from tailored strategies based on their individual risk profiles.

## Supplementary Material

ofaf532_Supplementary_Data

## Supplementary Data

Supplementary materials are available at *Open Forum Infectious Diseases* online. Consisting of data provided by the authors to benefit the reader, the posted materials are not copyedited and are the sole responsibility of the authors, so questions or comments should be addressed to the corresponding author.
