# Supplementary material for: Development of a Comprehensive Program for the Early Diagnosis and Treatment of Severe Infections in a Tertiary Hospital in Spain
Source: Open Forum Infect Dis. 2025 Sep 1;12(9):ofaf532. doi: 10.1093/ofid/ofaf532 (PMC12448488; doi:10.1093/ofid/ofaf532)
Supplement: ofaf532_Supplementary_Data [file ofaf532_supplementary_data.zip › Supplemental Material 2_OFID.docx]

**Supplementary Material 2**

Guillermo Martín-Gutiérrez, José Molina, Carlos Martín-Pérez, Manuela Aguilar-Guisado, María Solla-Fernández, Belén Ramos-Moreda, Teresa Aldabó, Rosario Amaya-Villar, Adelina Gimeno, Pilar Egea, Rocío Álvarez-Marín, José Antonio Lepe, José Miguel Cisneros. Development of a comprehensive program for the early diagnosis and treatment of severe infections in a tertiary hospital in Spain.

This supplementary material has been provided by the authors to offer additional methodological details and microbiological findings supporting the main manuscript. It includes relevant information on clinical microbiology workflows, diagnostic protocols, and microbiology results.

**Clinical Microbiology Procedures and Results**

***Microbiology laboratory methods***

In both the pre-intervention and intervention periods, blood culture bottles (Plus Aerobic/F and Lytic Anaerobic/F, Becton Dickinson) were incubated in a BacTec automated system (Becton Dickinson) until flagged as positive or for a maximum of 5 days. Gram staining was promptly performed on all positive bottles. In the pre-intervention period, direct identification from positive blood cultures was performed using MALDI-TOF (Bruker) as previously described (18). During the intervention period, the BCID panel (BioFire Diagnostics, bioMérieux) was used between 22:30 and 08:00 according to the manufacturer’s instructions. Identification and antimicrobial susceptibility testing were carried out using standard phenotypic methods and the MicroScan WalkAway system (Beckman Coulter, Inc., Brea, CA) in both study periods.

Regarding pneumonia diagnostics, during the pre-intervention period, standard-of-care included pneumococcal and *Legionella* urine antigen tests (Binax NOW, Alere) and a respiratory multiplex PCR for nasopharyngeal swabs targeting viral and atypical pathogens (Simplexa, DiaSorin Molecular). During the intervention period, this respiratory panel was extended to include SARS-CoV-2 detection (Vitro SA One-Step RT Kit) and was performed 24/7. In addition, BAL samples were tested using the BioFire FilmArray Pneumonia Plus panel (BioFire Diagnostics, bioMérieux), which detects 27 bacterial and viral pathogens along with 7 antimicrobial resistance genes (Supplementary Table 1). When fungal infections were suspected, the Aspergillus lateral flow device (AspLFD, OLM Diagnostics) and the eazyplex *Pneumocystis jirovecii* assay (AmplexDiagnostics) were employed.

***Results information***

During the pre-intervention period, the results were recorded in the patient’s electronic medical record. Blood cultures were informed by telephone call from 08:00 to 00:00. On the other hand, during the intervention period, the clinical staff managing patient care were informed of the results on a continuous 24/7 basis through direct communication with an infectious disease specialist available at all hours, and the test results were promptly documented in the patient’s electronic medical record. The report in the electronic medical record provided results, including standard responses suggesting whether the detected bacterium was a likely pathogen.

***Local antimicrobial resistance epidemiology***

During the study period, the prevalence of multidrug-resistant bacteria in our hospital was remarkably low. Only eight cases of extended-spectrum beta-lactamases (ESBLs) producing strains were detected (seven in patients with sepsis and one with pneumonia), and no strains with carbapenemase genes or vancomycin resistance mechanisms were isolated. For access to the cumulative antibiograms of our hospital, please visit the following website: https://www.guiaprioam.com/indice/antibiogramas-acumulados-de-total-de-muestras-excepto-ori

***Microbiological results***

Regarding microbiological results, a total of 85 (70.83%) patients with pneumonia had an etiological diagnosis, of which 62 (51.66%) were bacterial, 8 viral (6.66%) and 7 (5.83%) fungal infections. In addition, nine patients (7.5%) presented bacterial co-infections (produced by two different microorganisms), two patients (1.66%) presented viral and fungal co-infections, one patient (0.83%) presented viral and bacterial co-infection, and one patient (0.83%) a fungal co-infection produced by *Rhizopus orizae* and *Aspergillus flavus.* Of the 120 pneumonia cases included, 90 (75%) had a confirmed microbiological diagnosis, while 30 (25%) remained without an identified pathogen*.*No significant differences were found between the pre and the intervention periods (data not shown). Microbiological results in respiratory samples according to the type of pneumonia is summarized in **Supplemental Material – Table 2**. During the intervention period, syndromic respiratory panels for pneumonia pathogens were performed in 51 patients, of which 29 had a positive result (56.86%) and 22 a negative result (43.13%). Isothermal amplification for *P. jirovecii* was performed in 37 patients, of which two had a positive result (5.4%). *Aspergillus* lateral flow was performed on 58 patients, of which 10 were positive (17.24%) and 48 (92.75%) were negative (94.59%). In total, a rapid etiological diagnosis was reached in 37 patients (60.65%) (**Supplemental Material 2 – Table 3**).

With respect to the sepsis cohort, in 118 patients (54.1%) a positive result was obtained, and the microorganism responsible for the infectious syndrome was identified. The remaining 96 patients (44.03%) had a negative result, and in 6 cases (2.75%) the blood culture was contaminated with skin microbiota. (**Supplemental Material 2 – Table 4**).

| **Supplemental Material 2 – Table 1.** Molecular methods implemented during the intervention period for patients with pneumonia and sepsis | | | | | |
| --- | --- | --- | --- | --- | --- |
| **Pneumonia** | |  | **Sepsis** | | |
| **BIOFIRE® FILMARRAY® Pneumonia plus Panel** | |  | **BIOFIRE® FILMARRAY® 2 (BCID2) Panel** | | |
|  | Bacteria (semi quantitative) |  | Bacteria | | |
|  | *Acinetobacter calcoaceticus-baumannii complex* |  | *Acinetobacter calcoaceticus-baumannii complex* | | |
|  | *Enterobacter cloacae*complex |  | *Bacteroides fragilis* | | |
|  | *Escherichia coli* |  | *Enterobacterales* | | |
|  | *Haemophilus influenzae* |  |  | *Enterobacter cloacae complex* | |
|  | *Klebsiella aerogenes* |  |  | *Escherichia coli* | |
|  | *Klebsiella oxytoca* |  |  | *Klebsiella aerogenes* | |
|  | *Klebsiella pneumoniae*group |  |  | *Klebsiella oxytoca* | |
|  | *Moraxella catarrhalis* |  |  | *Klebsiella pneumoniae group* | |
|  | *Proteus*spp. |  |  | *Proteus spp.* | |
|  | *Pseudomonas aeruginosa* |  |  | *Salmonella spp.* | |
|  | *Serratia marcescens* |  |  | *Serratia marcescens* | |
|  | *Staphylococcus aureus* |  | *Haemophilus influenzae* | | |
|  | *Streptococcus agalactiae* |  | *Neisseria meningitidis* | | |
|  | *Streptococcus pneumoniae* |  | *Pseudomonas aeruginosa* | | |
|  | *Streptococcus pyogenes* |  | *Stenotrophomonas maltophilia* | | |
|  |  |  | *Enterococcus faecalis* | | |
| Atypical Bacteria (Qualitative) | |  | *Enterococcus faecium* | | |
|  | *Legionella pneumophila* |  | *Listeria monocytogenes* | | |
|  | *Mycoplasma pneumoniae* |  | *Staphylococcus spp.* | | |
|  | *Chlamydia pneumoniae* |  |  | *Staphylococcus aureus* | |
|  |  |  |  | *Staphylococcus epidermidis* | |
| Viruses | |  |  | *Staphylococcus lugdunensis* | |
|  | Adenovirus |  | *Streptococcus spp.* | | |
|  | Coronavirus |  |  | *Streptococcus agalactiae* | |
|  | Human metapneumovirus |  |  | *Streptococcus pneumoniae* | |
|  | Human rhinovirus/enterovirus |  |  | *Streptococcus pyogenes* | |
|  | Influenza A virus  Influenza B virus |  |  | Yeast | |
|  | Middle East respiratory syndrome coronavirus (MERS-CoV) |  |  | *Candida albicans* | |
|  | Parainfluenza virus |  |  | *Candida auris* | |
|  | Respiratory syncytial virus |  |  | *Candida glabrata* | |
|  |  |  |  | *Candida krusei* | |
| ESBL | |  |  | *Candida parapsilosis* | |
|  | CTX-M |  |  | *Candida tropicalis* | |
|  |  |  |  | *Cryptococcus (C. neoformans/C. gattii)* | |
| Carbapenemases | |  |  |  | |
|  | KPC |  | ESBL | | |
|  | NDM |  | CTX-M | |  |
|  | Oxa48-like |  |  |  | |
|  | VIM |  | Carbapenemases | | |
|  | IMP |  |  | KPC | |
|  |  |  |  | NDM | |
| Meticillin resistance | |  |  | Oxa48-like | |
|  | mecA/mecC and MREJ |  |  | VIM | |
|  |  |  |  | IMP | |
| **AAspergillus lateral flow assays (LFA-IMMY™)** | |  |  |  | |
|  |  | Meticillin resistance | | | |
| **Eazyplex Pneumocystis jirovecii kit** | |  | mecA/mecC and MREJ | | |

**Supplemental Material 2 – Table 2.** Microbiological detections in respiratory samples according to the type of pneumonia. N (%).

| **Result** | **CAP** | **COVID** | **Nosocomial (no VAP)** | **VAP** |
| --- | --- | --- | --- | --- |
| *Acinetobacter baumannii* | 0 | 0 | 0 | 2 (1,66%) |
| *Acinetobacter dijkshoorniae* | 0 | 0 | 0 | 1 (0,83%) |
| *Aspergillus fumigatus* | 0 | 1 (9,09%) | 0 | 2 (1,66%) |
| *Aspergillus lentulus* | 0 | 0 | 1 (3,22%) | 0 |
| *Aspergillus niger* | 0 | 0 | 1 (3,22%) | 1 (0,83%) |
| *Aspergillus terreus* | 0 | 0 | 1 (3,22%) | 0 |
| *Citrobacter freundii* | 0 | 0 | 0 | 1 (0,83%) |
| *Citrobacter koseri* | 0 | 0 | 0 | 1 (0,83%) |
| *Enterobacter cloacae* | 0 | 0 | 0 | 2 (1,66%) |
| *Enterococcus faecalis* | 0 | 0 | 0 | 1 (0,83%) |
| *Enterococcus faecium* | 0 | 0 | 1 (3,22%) | 0 |
| *Escherichia coli* | 0 | 0 | 0 | 7 (5,83%) |
| *Haemophilus influenzae* | 1 (5%) | 0 | 1 (3,22%) | 2 (1,66%) |
| *Haemophilus influenzae* + *Moraxella catarrhalis* | 0 | 0 | 0 | 1 (0,83%) |
| Influenza A | 1 (5%) | 0 | 1 (3,22%) | 0 |
| *Klebsiella pneumoniae* | 1 (5%) | 0 | 0 | 2 (1,66%) |
| *Legionella pneumophila* | 1 (5%) | 0 | 0 | 0 |
| *Parainfluenza virus* | 1 (5%) | 0 | 0 | 0 |
| *Pseudomonas aeruginosa* | 0 | 1 (9,09%) | 2 (6,45%) | 7 (5,83%) |
| *Pseudomonas aeruginosa + Haemophilus influenzae* | 0 | 0 | 0 | 1 (0,83%) |
| *Pseudomona aeruginosa +* Influenza A | 0 | 0 | 0 | 1 (0,83%) |
| *Rhizopus orizae + Aspergillus flavus* | 0 | 0 | 1 (3,22%) | 0 |
| *Serratia marcescens* | 0 | 0 | 0 | 3 (2,5%) |
| *Staphylococcus aureus* | 0 | 1 (9,09%) | 1 (3,22%) | 4 (3,33%) |
| *Staphylococcus aureus + Haemophilus influenzae* | 0 | 0 | 0 | 1 (0,83%) |
| *Staphylococcus aureus* + *Klebsiella pneumoniae* | 0 | 0 | 1 (3,22%) | 1 (0,83%) |
| *Staphylococcus aureus* + *Pseudomonas aeruginosa* | 0 | 0 | 0 | 1 (0,83%) |
| *Stenotrophomonas maltophilia* | 0 | 0 | 0 | 1 (0,83%) |
| *Streptococcus pneumoniae* | 2 (10%) | 0 | 0 | 0 |
| *Streptococcus pneumoniae + Haemophilus influenzae* | 1 (5%) | 0 | 1 (3,22%) | 0 |
| Normal bacterial flora | 2 (10%) | 4 (36,36%) | 2 (6,45%) | 4 (3,33%) |
| Culture negative | 10 (50%) | 4 (36,36%) | 17 (54,83%) | 9 (7,5%) |
| Total | 20 (16,66%) | 11 (9,16%) | 31 (25,83%) | 56 (46,66%) |

**Supplemental Material 2 – Table 3.** Results obtained using rapid diagnostic methods in the intervention period. LFA: Galactomannan Lateral Flow Assay.

| **Results** | **N** | **%** |
| --- | --- | --- |
| *Acinetobacter calcoaceticus-baumannii complex* | 1 | 1,64 |
| CMV | 1 | 1,64 |
| *Escherichia coli* | 2 | 3,28 |
| *Escherichia coli + COVID + Pneumocystis jirovecii + galactomanano* | 1 | 1,64 |
| *Escherichia coli + Moraxella catarrhalis + Streptococcus pneumoniae* | 1 | 1,64 |
| *Escherichia coli + Moraxella catarrhalis + Staphylococcus aureus* | 1 | 1,64 |
| *Escherichia coli + Streptococcus peumoniae* | 1 | 1,64 |
| Positive LFA | 7 | 11,48 |
| *Haemophilus influenzae* | 2 | 3,28 |
| *Haemophilus influenzae + galactomanano* | 1 | 1,64 |
| *Haemophilus influenzae + Streptococcus pneumoniae* | 1 | 1,64 |
| *Klebsiella pneumoniae* | 2 | 3,28 |
| *Legionella pneumophila* | 1 | 1,64 |
| *Moraxella catarrhalis + Streptococcus pneumoniae + VRS* | 1 | 1,64 |
| Parainfluenza virus | 1 | 1,64 |
| *Pneumocistis jirovecii + galactaomanano* | 1 | 1,64 |
| *Pseudomonas aeruginosa* | 3 | 4,92 |
| *Pseudomonas aeruginosa + Staphylococcus aureus* | 1 | 1,64 |
| *Serratia marcescens* | 2 | 3,28 |
| *Staphylococcus aureus meticilin sensible* | 1 | 1,64 |
| *Streptococcus pneumoniae + Moraxella catarralis* | 1 | 1,64 |
| *Streptococcus pneumoniae + Staphylococcus aureus* | 1 | 1,64 |
| Influenza virus B | 2 | 1,64 |
| VRS | 1 | 1,64 |
| Negative result | 24 | 37,70 |

| **Supplemental Material 2 – Table 4.** Microbiological detections in blood cultures. | | |
| --- | --- | --- |
| **Microorganism** | **N** | **%** |
| *Escherichia coli* | 33 | 15,14 |
| *Klebsiella pneumoniae* | 19 | 8,72 |
| *Staphylococcus aureus* | 13 | 5.96 |
| *Pseudomonas aeruginosa* | 10 | 4.59 |
| *Enterobacter cloacae* | 4 | 1.83 |
| *Streptococcus pneumoniae* | 3 | 1.38 |
| *Candida albicans* | 2 | 0.92 |
| *Clostridium perfringens* | 2 | 0.92 |
| *Proteus mirabilis* | 2 | 0.92 |
| *Streptococcus anginosus* | 2 | 0.92 |
| *Acinetobacter baumannii* | 1 | 0.46 |
| *Aeromonas caviae + Klebsiella oxytoca* | 1 | 0.46 |
| *Bacteroides fragilis* | 1 | 0.46 |
| *Bacteroides thetaiotaomicron* | 1 | 0.46 |
| *Bacteroides vulgatus* | 1 | 0.46 |
| *Candida lusitaniae* | 1 | 0.46 |
| *Candida parapsilosis* | 1 | 0.46 |
| *Candida tropicalis* | 1 | 0.46 |
| *Capnocytophaga canimorsus* | 1 | 0.46 |
| *Carnobacterium divergens* | 1 | 0.46 |
| *Citrobacter freundii + Enterococcus faecalis* | 1 | 0.46 |
| *Enterococcus faecalis* | 1 | 0.46 |
| *Enterococcus faecalis + Staphylococcus aureus* | 1 | 0.46 |
| *Enterococcus faecium* | 1 | 0.46 |
| *Escherichia coli + Klebsiella pneumoniae* | 1 | 0.46 |
| *Escherichia coli + Staphylococcus aureus* | 1 | 0.46 |
| *Haemophilus influenzae* | 1 | 0.46 |
| *Haemophilus parainfluenzae* | 1 | 0.46 |
| *Klebsiella oxytoca* | 1 | 0.46 |
| *Moraxella catarrhalis* | 1 | 0.46 |
| *Providencia stuartii* | 1 | 0.46 |
| *Pseudoglutamicibacter cumminsii* | 1 | 0.46 |
| *Pseudomonas aeruginosa + Citrobacter amalonaticus* | 1 | 0.46 |
| *Pseudomonas putida* | 1 | 0.46 |
| *Staphylococcus lugdunensis* | 1 | 0.46 |
| *Streptococcus agalactiae* | 1 | 0.46 |
| *Streptococcus oralis* | 1 | 0.46 |
| *Streptococcus parasanguinis* | 1 | 0.46 |
| *Streptococcus pyogenes* | 1 | 0.46 |
