# Supplementary material for: Development of a Comprehensive Program for the Early Diagnosis and Treatment of Severe Infections in a Tertiary Hospital in Spain
Source: Open Forum Infect Dis. 2025 Sep 1;12(9):ofaf532. doi: 10.1093/ofid/ofaf532 (PMC12448488; doi:10.1093/ofid/ofaf532)
Supplement: ofaf532_Supplementary_Data [file ofaf532_supplementary_data.zip › Supplemental Material 1_OFID.docx]

**Supplemental Material 1**

Guillermo Martín-Gutiérrez, José Molina, Carlos Martín-Pérez, Manuela Aguilar-Guisado, María Solla-Fernández, Belén Ramos-Moreda, Teresa Aldabó, Rosario Amaya-Villar, Adelina Gimeno, Pilar Egea, Rocío Álvarez-Marín, José Antonio Lepe, José Miguel Cisneros. Development of a comprehensive program for the early diagnosis and treatment of severe infections in a tertiary hospital in Spain.

This supplemental material has been provided by the authors to give readers additional information about their work. Contents are presented in the order that appeared in the text.

**Supplementary table 1.** Definitions and diagnostic clinical criteria for severe pneumonia.

**Supplementary Table 2.** DOOR-MAT analysis for patients with aetiological bacterial diagnosis.

**Supplementary Table 3**. Multivariable analysis for potential factors associated with length of stay.

**Supplementary Table 4.** Multivariable Cox regression model with all variables identified in the bivariate analysis related to mortality.

**Supplementary Figure 1**.

**Supplementary Figure 2.**

**Supplementary Figure 3.**

**Supplementary Figure 4.**

**Supplementary Figure 5.**

| **Supplementary table S1**. Definitions and diagnostic clinical criteria of severe pneumonia. | |
| --- | --- |
| **Type of pneumonia** | **Diagnostic criteria** |
| Clinical CAP (1) | • Cough: recent or worsening, production of purulent sputum or a change in the character of sputum. |
|  | • Temperature >38.0°C or <36.1°C. |
|  | • Auscultatory findings consistent with pneumonia, including rales, evidence of pulmonary consolidation (dullness on percussion, bronchial breath sounds, or egophony), or both. |
|  | • Leukocytosis (>11.0 × 109 white cells per litter or > 8.2 x 109 neutrophils), C-reactive protein (CRP) level > 50 mg/L. |
|  | • Dyspnea, tachypnoea, or hypoxemia: recent or worsening. |
|  | • CURB65 score ≥2 (2). |
| Nosocomial pneumonia (3,4) | • Worsening oxygenation, defined as at least 2 days of i) decrease in daily minimum oxygen saturation, ii) initiation of supplemental oxygen or iii) escalation of supplemental oxygen. |
|  | • Either temperature higher than 38ºC and/or i) leukocytosis (≥12,000 cells/mm^3^) or leukopenia (≤4000 cells/mm^3^).  • Classic signs and symptoms compatible with lung infection also include new onset cough, fever, shortness of breath, pleuritic chest pain, and bronchial breath sounds.  • Performance of chest imaging. |
| VAP (5) | • Sustained increase in oxygen requirements in a ventilated patient over a period of 2 days. Sustained oxygen requirement is defined as an increase in the daily minimum positive end-expiratory pressure (PEEP) of greater than or equal to 3 cm H2O or an increase in the daily minimum fraction of inspired oxygen (Fio 2) of greater than or equal to 20 points for 2 days. |
|  | • The patient must have had a minimum of 2 days of mechanical ventilation with stable or decreasing oxygen requirements before the days of increased oxygenation. |
|  | • Low-grade fever (>38°C) or hypothermia (<36°C). |
|  | • Leukocytosis (≥12,000 cells/mm3) or leukopenia (≤4000 cells/mm3). |
|  | • Purulent respiratory secretions, defined as secretions form the lungs, bronchi, or trachea that contain >25 neutrophils and < 10 squamous epithelial cells per low power filed (x10). |
| CAP: community acquired pneumonia; VAP: ventilator-associated pneumonia; ICPs: immunocompromised patients. | |

References:

1. Metlay JP et al. Diagnosis and Treatment of Adults with Community-acquired Pneumonia. An Official Clinical Practice Guideline of the American Thoracic Society and Infectious Diseases Society of America. Am J Respir Crit Care Med. 2019; 200:e45-e67
2. Lim WS et al. Defining community acquired pneumonia severity on presentation to hospital: an international derivation and validationstudy. *Thorax.* 2023; 58:377–82.
3. Cheng GS et al. Immunocompromised Host Pneumonia: Definitions and Diagnostic Criteria: An Official American Thoracic Society Workshop Report. Ann Am Thorac Soc. 2023; 20:341-353.
4. Ji W, McKenna C, Ochoa A, et al. Development and Assessment of Objective Surveillance Definitions for Nonventilator Hospital-Acquired Pneumonia. *JAMA Netw Open*. 2019;2(10):e1913674. Published 2019 Oct 2. doi:10.1001/jamanetworkopen.2019.13674
5. National Healthcare Safety Network (NHSN) July 2013 CDC/NHSN Protocol Clarifications 2013. [[http://www.cdc.gov/nhsn/PDFs/pscManual/10-VAE_FINAL.](http://www.cdc.gov/nhsn/PDFs/pscManual/10-VAE_FINAL.pdf)

**Supplementary Table 2**. DOOR-MAT analysis for patients with aetiological bacterial diagnosis. N (%)

| **A** | | | | | | | | | |  |
| --- | --- | --- | --- | --- | --- | --- | --- | --- | --- | --- |
|  | | **Empirical treatment** | | | **Pathogen-directed treatment** | | | **Treatment after final report** | | |
|  |  | **Pre-interv** | **Intervention** | ***p-*value** | **Pre-interv** | **Intervention** | ***p-*value** | **Pre-interv** | **Intervention** | ***p-*value** |
| **Antimicrobial spectrum** | 0 | 0 (0) | 1 (2.9) | 0.187 | 2 (5.6) | 0 (0) | 0.264 | 0 (0) | 3 (11.5) | 0.152 |
|  | I | 5 (11.6) | 1 (2.9) |  | 2 (5.6) | 2 (7.1) |  | 6 (17.1) | 4 (15.4) |  |
|  | II | 5 (11.6) | 3 (8.8) |  | 4 (11.1) | 4 (14.3) |  | 1 (2.9) | 0 (0) |  |
|  | III | 30 (69.8) | 18 (52.9) |  | 26 (72.2) | 26 (50) |  | 25 (71.4) | 12 (46.2) |  |
|  | IV | 2 (4.7) | 11 (32.4) |  | 2 (5.6) | 8 (28.6) |  | 2 (5.7) | 6 (23.1) |  |
|  | V | 1 (2.3) | 0 (0) |  | 0 (0) | 0 (0) |  | 1 (2.9) | 1 (3.8) |  |
| **DOOR-MAT score**  **(Mean [STD])^a^** | | - | - | - | 74.2 (31.2) | 81.2 (27.8) | 0.213 | 93.7 (8.9) | 94.1 (7.8) | 0.5579 |
| Pre-interv: pre-intervention period. ^a^ The DOOR-MAT score was not calculated for empirical treatment, as it was not based on any aetiological identification^.^ | | | | | | | | | | |
| **B** | | | | | | | | | |  |
|  | | **Empirical treatment** | | | **Pathogen-directed treatment** | | | **Treatment after final report** | | |
|  |  | **Pre-interv** | **Intervention** | ***p-*value** | **Pre-interv** | **Intervention** | ***p-*value** | **Pre-interv** | **Intervention** | ***p-*value** |
| **Antimicrobial spectrum** | 0 | 1 (2) | 0 (0) | 0.589 | 1 (2) | 0 (0) | 0.499 | 0 (0) | 0 (0) | 0.201 |
|  | I | 0 (0) | 1 (2) |  | 2 (4) | 1 (2) |  | 8 (13) | 2 (4) |  |
|  | II | 1 (2) | 3 (6) |  | 3 (5) | 6 (12) |  | 9 (14) | 3 (6) |  |
|  | III | 46 (72) | 37 (69) |  | 42 (65) | 31 (57) |  | 37 (59) | 39 (72.2) |  |
|  | IV | 14 (2) | 10 (19) |  | 15 (25) | 14 (29) |  | 9 (14) | 8 (17) |  |
|  | V | 2 (3) | 3 (6) |  | 1 (2) | 2 (4) |  | 0 (0) | 1 (2) |  |
| **DOOR-MAT score**  **(Mean [STD])^a^** | | - | - | - | 71.2 (24.5) | 81.2 (20.4) | **0.002** | 80.4 (19.5) | 88.0 (10.8) | **0.024** |
| Pre-interv: pre-intervention period. ^a^ The DOOR-MAT score was not calculated for empirical treatment, as it was not based on any aetiological identification^.^  A. Patients with severe pneumonia. B. Patients with sepsis | | | | | | | | | | |

| **Supplementary Table 3**. Multivariable analysis for potential factors associated with length of stay in patients with pneumonia. | | | | |
| --- | --- | --- | --- | --- |
| **Variable** | **β** | **Standard error** | **t-value** | ***p-*value** |
| Drinking history | 465.8 | 174.9 | 2.66 | **0.009** |
| VAP | 375.8 | 155.7 | 2.41 | **0.018** |
| Molecular diagnosis | -407.9 | 172.8 | -2.361 | **0.021** |
| β: regression coefficient; t-value: β divided by the standard error. | | | | |

| **Supplementary Table 4**. Multivariable Cox regression model with all variables identified in the bivariate analysis related with mortality. | | | | | | | | |  |
| --- | --- | --- | --- | --- | --- | --- | --- | --- | --- |
|  |  |  |  |  |  |  |  |  |  |
| **Pneumonia** | | | |  | **Sepsis** | | | |  |
| Variable | **se β** | **HR** | **p-Value** |  | **Variable** | **se β** | **HR** | **p-Value** |  |
| Fungal infection | 1.115 | 3.049 | **0.007** |  | Etiological diagnosis | -3.001 | 0.049 | **0.006** |  |
| Admissions due to influenza infection | 1.135 | 3.112 | **0.008** |  | Change of treatment | -1.633 | 0.195 | **<0.001** |  |
| Charlson Index | 0.154 | 1.167 | **0.036** |  | DOOR-MAT^a^ | 0.03 | 0.97 | **0.002** |  |
|  |  |  |  |  | Charlson Index | 0.289 | 1.335 | **0.009** |  |
|  |  |  |  |  | Time to empirical treatment | 1.577 | 4.840 | **0.043** |  |
| se β, regression coefficient. HR, hazard ratio.  ^a^dDOOR-MAT score after final microbiological report. | | | | | | | | |  |

**
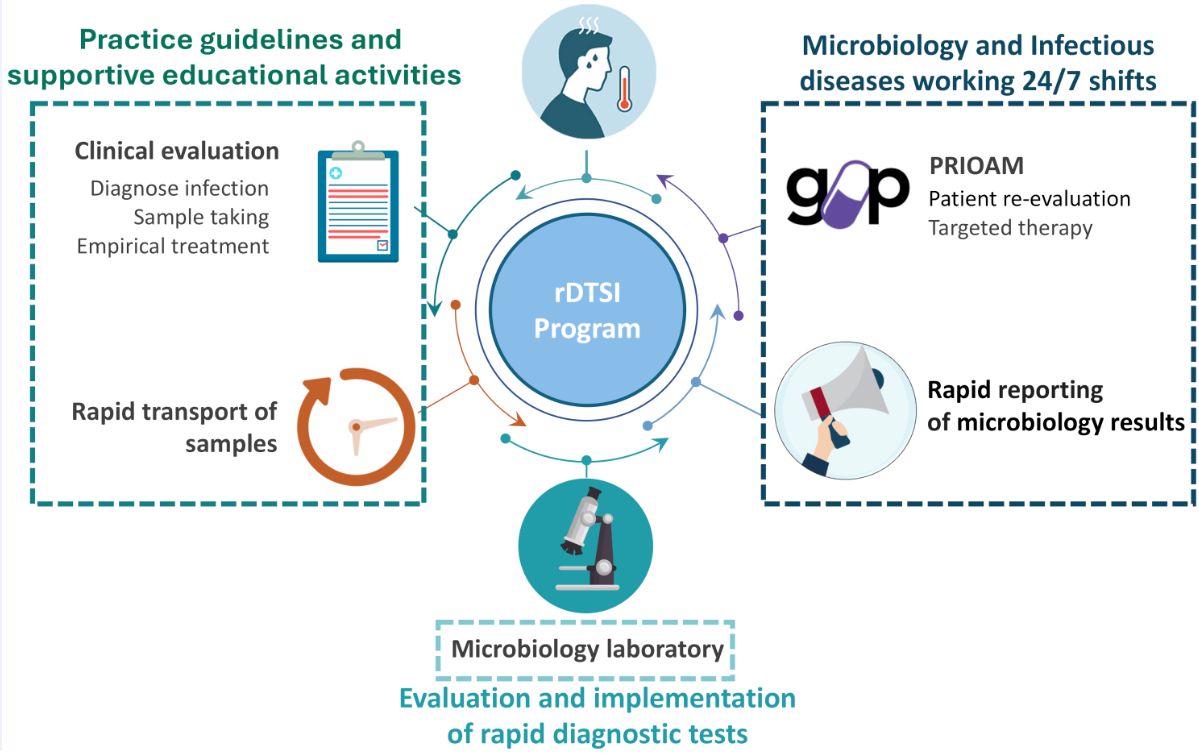
**

**Supplementary Figure 1**. Summary of activities conducted in the rDTSI programme. This material was designed to ensure consistent understanding and implementation across all teams involved in the project. This figure has been designed using images from Freepik ([www.freepik.es](http://www.freepik.es)).

**
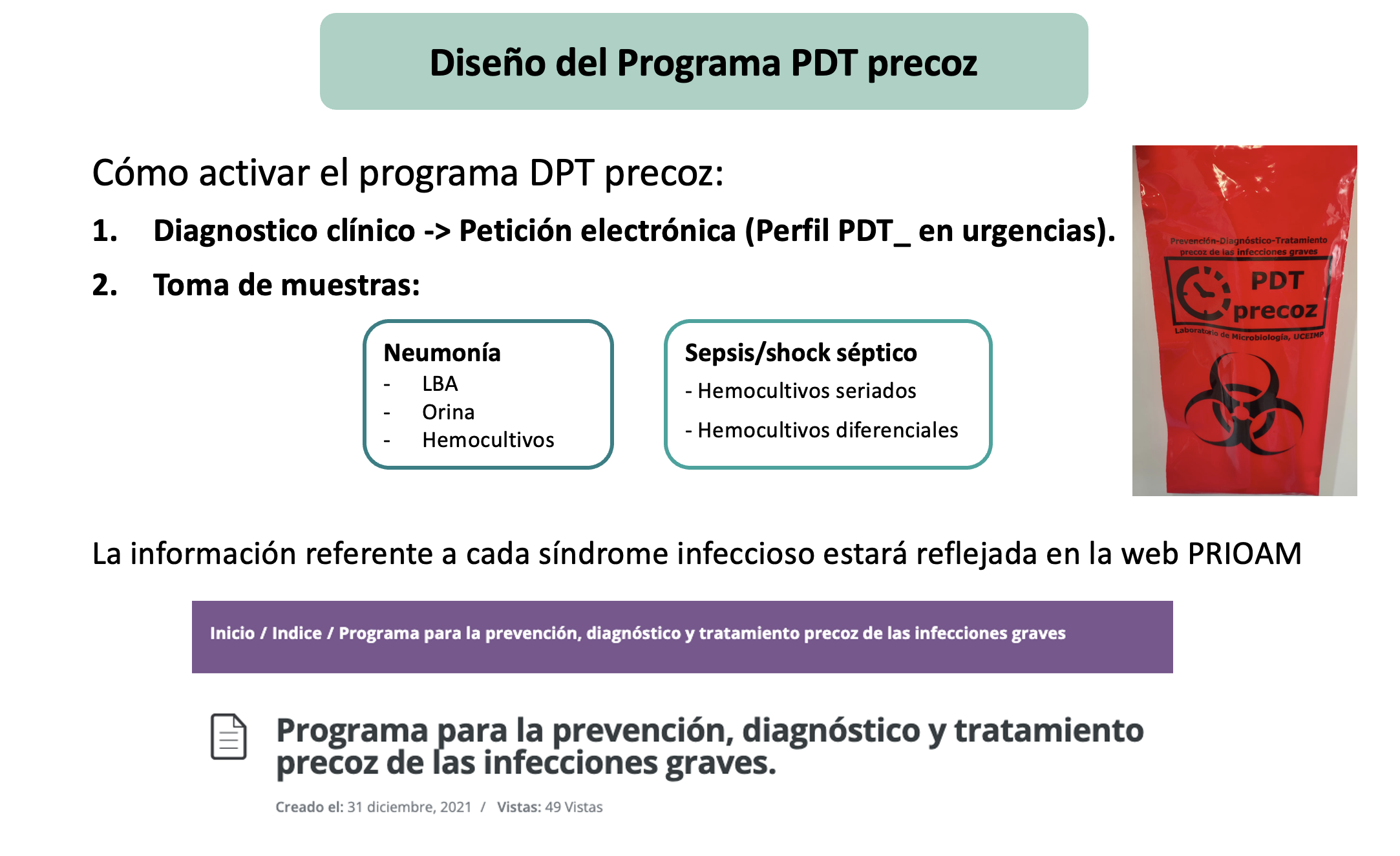
**

**Supplementary Figure 2**. Example of the informational material provided to clinical teams for training purposes, illustrating the steps and key procedures required to activate the program. The image includes the red transport bag, specifically designated for urgent samples, to differentiate them from other types of samples and ensure prioritized handling. This material was designed to ensure consistent understanding and implementation across all teams involved in the project.

**
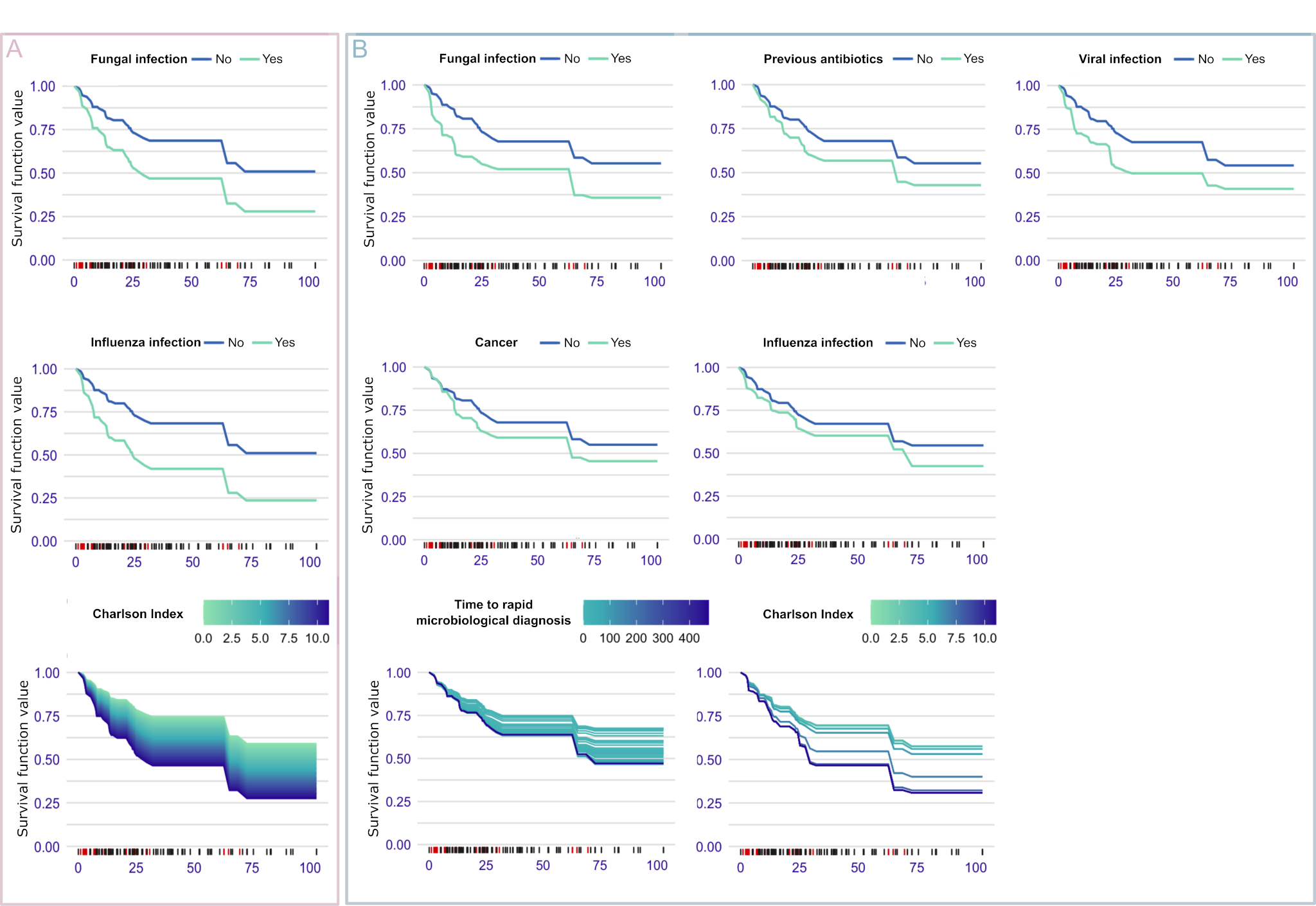
**

**Supplementary Figure 3.** Partial dependence survival profiles interpreting the effects of predictor variables in both Cox regression (A) and RSF models (B) in patients with severe pneumonia.

**A**


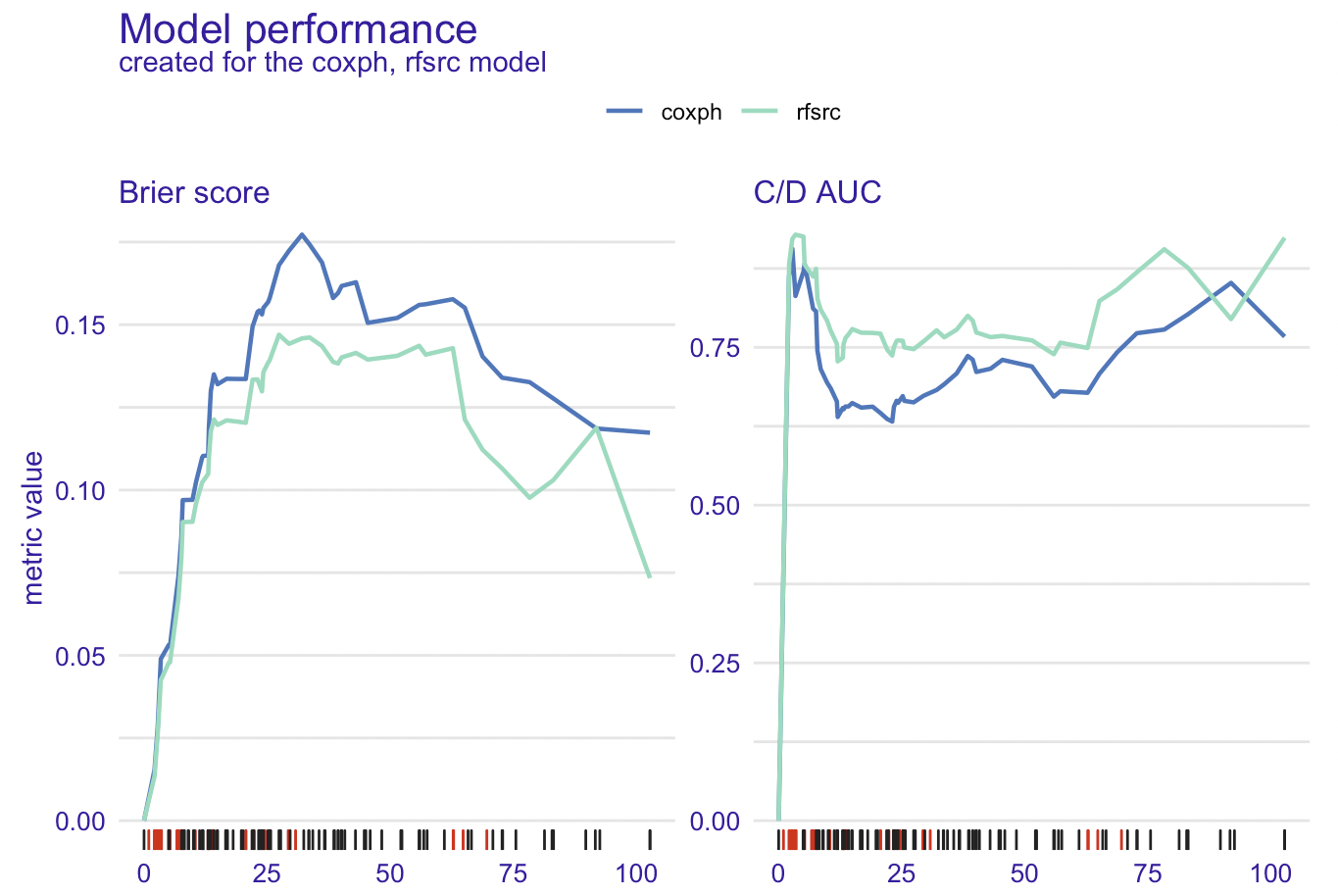


**B**


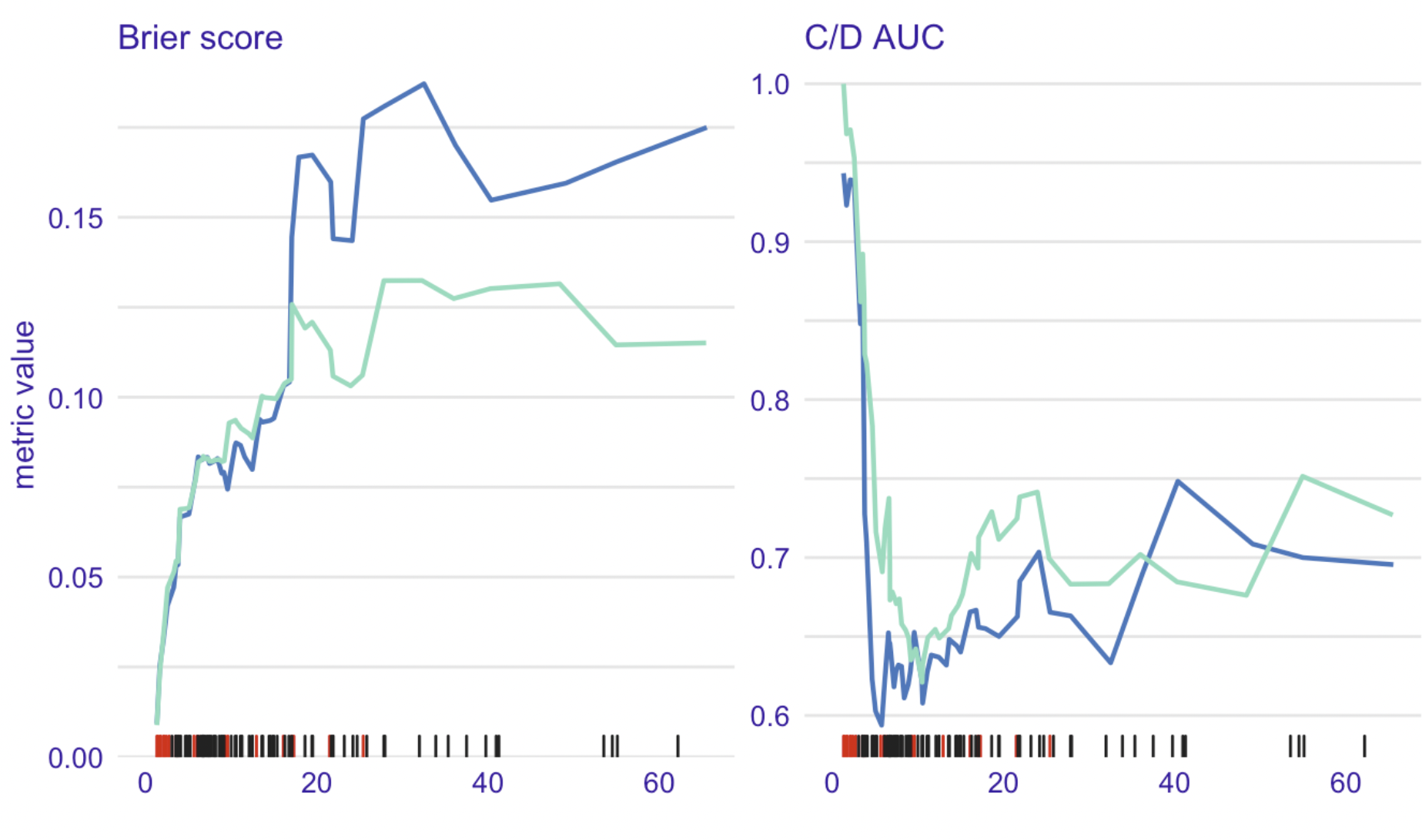


**Supplementary Figure 4.** Brier score and the cumulative/dynamic AUC for pneumonia (A) and sepsis (B) models.

**
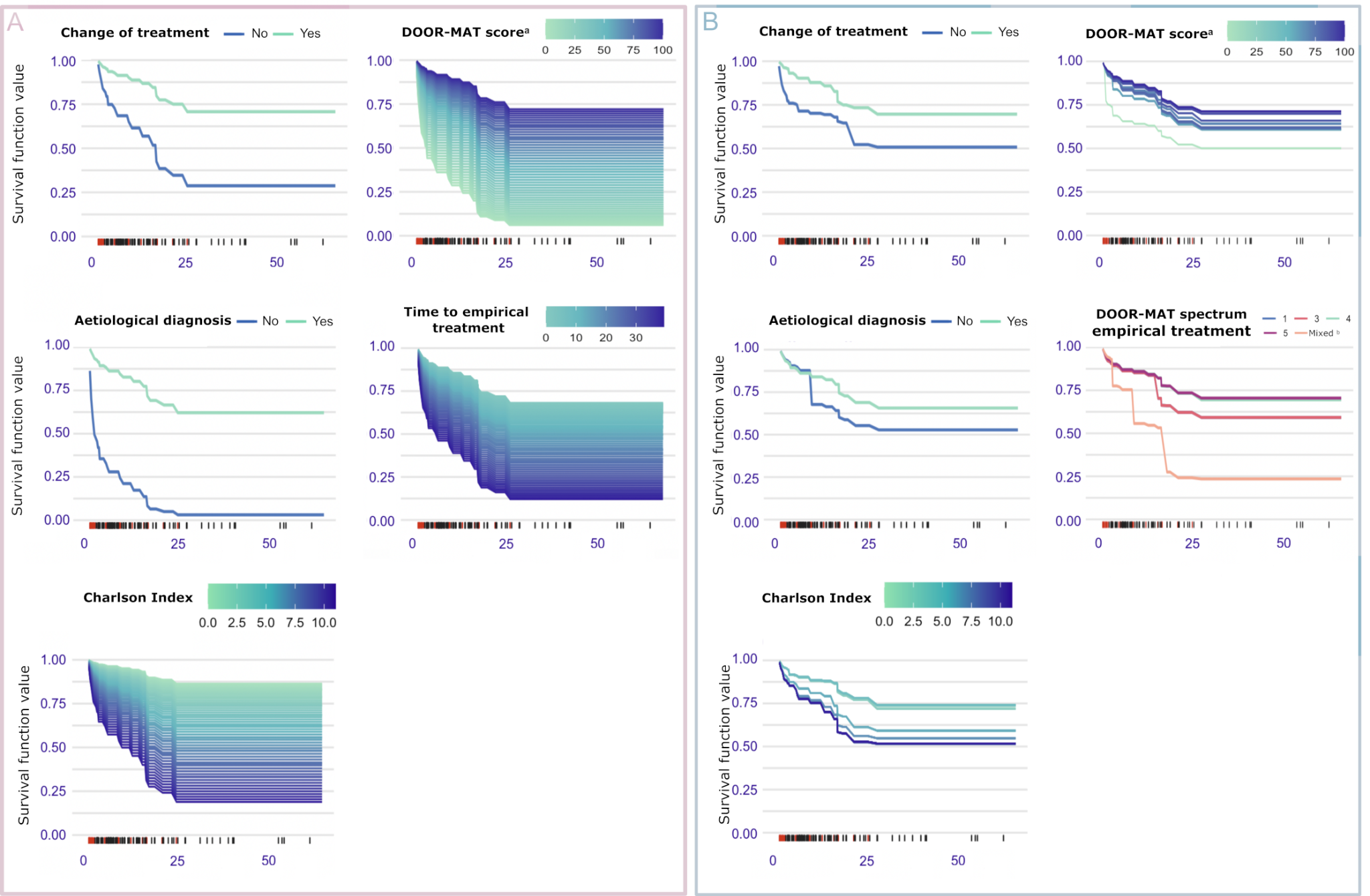
**

**Supplementary Figure 5.** Partial dependence survival profiles interpreting the effects of predictor variables in both Cox regression (A) and RSF models (B) in patients with sepsis.
